# Basic and clinical genetic studies on male infertility in Iran during 2000-2016: A review

**Published:** 2018-03

**Authors:** Sahar Moghbelinejad, Hossein Mozdarani, Pegah Ghoraeian, Reihaneh Asadi

**Affiliations:** 1 *Department of Genetics, School of Sciences, Qazvin University of Medical Sciences, Qazvin, Iran.*; 2 *Department of Medical Genetics, Faculty of Medical Sciences, Tarbiat Modares University, Tehran, Iran.*; 3 *Tehran Medical Sciences Branch, Islamic Azad University, Tehran, Iran.*; 4 *Cellular and Molecular Research Center, Research Institute for Endocrine Sciences, Shahid Beheshti University of Medical Sciences, Tehran, Iran.*

**Keywords:** Iran, Male infertility, Genetics, Cytogenetic, Peripheral blood, Sperm

## Abstract

The male factor contributes to 50% of infertility. The cause of male infertility is idiopathic and could be congenital or acquired. Among different factors which are involved in idiopathic male infertility, genetic factors are the most prevalent causes of the disease. Considering, the high prevalence of male infertility in Iran and the importance of genetic factors in the accession of it, in this article we reviewed the various studies which have been published during the last 17 yr on the genetic basis of male infertility in Iran. To do this, the PubMed and Scientific information database (SID) were regarded for the most relevant papers published in the last 17 yr referring to the genetics of male factor infertility using the keywords ‘‘genetics’’, “cytogenetic”, ‘‘male infertility”, and “Iranian population”. Literatures showed that among the Iranian infertile men Yq microdeletion and chromosomal aberrations are two main factors that intervene in the genetics of male infertility. Also, protamine deficiency (especially P2) is shown to have an influence on fertilization rate and pregnancy outcomes. The highest rate of sperm DNA damages has been found among the asthenospermia patients. In several papers, the relation between other important factors such as single gene mutations and polymorphisms with male infertility has also been reported. Recognition of the genetic factors that influence the fertility of Iranian men will shed light on the creation of guidelines for the diagnosis, consultation, and treatment of the patients."

## Introduction

Although there have been great achievements in medical sciences especially in reproductive sciences, infertility is still one of the major concern for young couples. Normally, 80-85% of unprotected intercourses in couples intending to have kids will end in pregnancy. It is estimated that 15% of couples attempting their first pregnancy may experience difficulty in conceiving. Infertility is actually a multifactorial problem in which different genetics, environmental, and anatomic factors can be influential. Studies have shown that 50% of infertilities are related to men (1). Several reasons were proposed for male infertility including anti-sperm antibody production, defective delivery of sperm, obstruction of seminal tract, etc.; among these, 40-90% of male infertilities are said to be related to impaired spermatogenesis, which is a significant rate (2). Semen analysis of men suffering from impaired spermatogenesis shows abnormal semen parameters manifested as azoospermia, oligozoospermia, theratozoospermia, and asthenozoospermia. Among these, genetic factors are the most important factors in male infertility, affecting wide physiological processes such as hormonal homeostasis, spermatogenesis, and quality of sperms (1). Up to now, about 200 genes have been detected which can control spermatogenesis, among which 30 genes are located on Y chromosome. Two important genetics factors in men infertilities are Y chromosome microdeletions and chromosomal abnormalities; although there are other factors such as protamine deficiency, sperm DNA damage, single gene mutation, etc. Knowing this fact and considering eminent advances in treating infertility by using assisted reproductive technique (ART), such as in vitro fertilization (IVF) and intra-cytoplasmic sperm injection (ICSI) which increase the possibility of transition of genetic abnormalities to next generations, detection of these genetics abnormalities would be valuable, in order to help infertile couples (3, 4). Infertility in Iran is also known as one of the major concerns of young couples. It is estimated that around 20.2 % of Iranian couples are infertile which is far more than the universal rate (12-15%); 70% of infertilities were reported to be related to male factor (3). Since the genetics is one of the critical factors causing male infertility, in this article we are going to review the researches done in Iran on the genetics of male infertility.

## Chromosome abnormality studies

The prevalence of chromosome abnormalities including numerical and structural aberrations (balanced and robertsonian translocations, duplications, deletions, inversions, etc.) in infertile men has been reported to be between 2-16% (5) which is much higher than the general population with the frequency around 0.6% (6). The incidence of chromosomal aberrations increase with the severity of infertility; this has been reported to be 6% in oligozoospermia and 14% in non-obstructive azoospermic (NOA) males (7). Consequently, chromosome aberrations analysis (karyotyping) is recommended as part of the infertility workup, if the infertile couple had experienced repeated spontaneous abortions or the semen analysis of the male partner was abnormal. In this case, several studies have been performed in order to ascertain the occurrence of chromosomal aberrations among Iranian males suffering from infertility. Reviewing the literature on the prevalence of chromosomal abnormalities among Iranian infertile men has indicated different frequencies ranging from 3.6-16.7% (8-13) which is within the range of chromosomal aberrations in infertile men reported worldwide, except for a paper published by Azimi and colleagues, in which they found out a much higher frequency of chromosomal aberrations (32.81%) among the patients. The authors believed that the highly selected patients-group, in their research might be responsible for this discrepancy (12). In studies in which the patients were categorized according to the semen parameters, the highest frequency of abnormal karyotypes, as expected, was found among patients with azoospermia, which ranged from 10-20% (10-13).

Since sex chromosome aberrations (numerical or structural alteration in X and Y chromosomes) are the most prevalent chromosome-related causes of infertility studying the frequency of these anomalies in infertile patients would be valuable; because by having these data physicians or genetics counselors can decide whether it is needed to order a cytogenetic test or not, which can help to save both time and money. Regarding the results of papers, 47,XXY, Klinefelter syndrome (KFS) was found to be the most frequent chromosomal anomaly (8.5-33.5%) among Iranian infertile men (8-12); while Salahshourifar and coworkers detected only one patient with KFS (0.1%) among 863 infertile men (6). In another paper, the frequency of sex chromosome aneuploidy (SCA) in blood samples of patients (male and female) from south of Iran was evaluated by Jouyan and coworkers (10). Their results suggested that 5.54% of cases have chromosomally abnormal karyotype, from which 30% was found to have SCA, including 46% of Turner’s syndrome and 46% of KFS and the remaining, other sex chromosome abnormalities. The rate of SCA in this study was lower than what was expected. It was mentioned that this discrepancy might be due to different factors such as genetics or environmental background.

One of the structural chromosome aberrations which frequently occur is the pericentric inversion of the chromosome 9. Although this abnormality is usually considered as a normal variant, an association of this inversion with subfertility, recurrent spontaneous abortions, and abnormal clinical phenotypes has been reported by several authors (14-17).

In this case, Khaleghian and Azimi have reported a rare case with homozygosity for pericentric inversions of chromosome 9 in a woman with 28 wk stillbirth, while both of her parents were heterozygotes for the inversions of chromosome 9 (16). Since these authors did not show any phenotypic abnormality, it was not clear whether the chromosome 9 inversion were responsible for the stillbirth or not. On the other hand, Mozdarani and colleagues have proposed a possible relationship between male infertility and inversion of chromosome 9 (17). After chromosomal analysis of 300 infertile couples, these investigators showed a total frequency of 2.5% for chromosome 9 inversion, which was higher compared to normal population and even to female patients (17). In 2013, Ghazaey *et al* showed a pericentric inversion of chromosome 9 in 5% of patients with recurrent spontaneous abortion (RSA) (11).

ART is used to circumvent human infertility. Several studies have shown different ranges of chromosomal abnormalities in infertile couples attending ART (18). In order to evaluate the prevalence and the type of these chromosomal abnormalities in Iranian couples who were candidates for assisted reproductive techniques, Salahshourifar and coworkers performed a cytogenetic analysis on the blood samples of 1726 candidate patients (863 men and 863 women) which revealed a total of 107 aberrant karyotypes (6.2%) (7). The frequencies of total abnormalities were 3.6% for men and 8.8% for women. There was also seen a high frequency of chromosomal abnormalities among couples with reproductive failure and a higher frequency of aberrations in women compared to men. Although the frequency of translocations was similar in both sexes, but the incidence of inversions was higher in men compared to women. Sex chromosome mosaicism in women was found to be higher than men (7).

ICSI is an efficient procedure which allows an infertile couple, especially in severe male factor cases to overcome their infertility. But regarding a threefold increased risk of sperm aneuploidy in infertile men and even up to 10-fold in the case of severe infertilities (19, 20) which might transmit to the next generation, there is a great concern about a possible increased risk of producing aneuploid embryos, as well as aneuploid offspring, particularly for sex chromosomes (21). Consequently, the direct analysis of the human gametes nuclei would be valuable in order to evaluate the occurrence of these abnormalities. In this case, spermatozoa of normal and infertile men have been studied cytogenetically by Hamster oocyte penetration assay, and further by molecular cytogenetic methods such as fluorescence in situ hybridization (FISH) and Primed in situ labelling (PRINS) assays (22, 23). In Iran, the first cytogenetic studies on spermatozoa were presented by Mozdarani and Aghdaei, in which the incidence of sperm premature chromosome condensation (PCC) in the failed fertilized oocytes following IVF and ICSI procedure was evaluated (24, 25). The results illustrated a high frequency of intact sperm head and sperm PCC in the failed fertilized oocytes, following Giemsa staining. On the other hand, the numbers of intact sperm heads, as well as PCC of the sperm, were higher in ICSI procedure compared to IVF. They concluded that other factors such as the failure of oocyte activation or an immature retrieval of oocytes were more related to above abnormalities than sperm anomalies (25). 

Mohseni and Mozdarani presented the first study in Iran, which applied zona-free hamster oocytes in order to investigate the frequency of sperm PCC induction among the Iranian normal and infertile men (astheno and oligospermic patients) (26). Their results showed much higher frequency of sperm PCC in asthenospermic samples, compared to sperm from oligospermic or normal men. Considering these results, in individuals with sperm abnormalities particularly in asthenospermic men, sperm PCC was mentioned as a major cause of fertilization failure and some idiopathic infertile. In another study done by the same authors (27), in addition to PCC, the frequency of numerical chromosome abnormalities in the sperm of normal and sub-fertile (oligospermic) men were evaluated, using zona-free hamster oocytes. They reported a higher frequency of PCC as well as numerical chromosome abnormalities in infertile patients compared to the normal group. Although in the case of numerical aberrations the differences were not significant, it was concluded that sperm numerical chromosome abnormalities were involved in male infertility. In another study, the rate of fertilization and PCC formation was evaluated after ICSI of hamster oocytes with irradiated sperms from normal and oligosperm individuals. This study showed fertilization rate and frequency of PCC in failed fertilized oocytes was significantly higher in oligospermic patients compared with normal ones (28). The possible cause of precluding oocytes from fertilization in oligospermic individuals was supposed to be due to the formation of PCC (28).

Considering the importance of chromosomal aneuploidy in male infertility, particularly for sex chromosomes, Ghoraeian *et al* studied the possible impact of sex chromosomal aneuploidy in spermatozoa on fertilization and implantation rate after ICSI (29). This was the first study done in Iran, which applied PRINS technique in order to assess the frequency of X and Y disomy in sperm samples retrieved from normal and oligozoospermic men (29). In this case, following ICSI, the rate of eight cell embryos in each group was determined and followed up for successful implantation. Regarding the results, a significantly higher rate of sperm sex chromosomes disomy and a lower rate of embryo formation were observed in oligozoospermic patients compared to normal men. Also, implantation rate for oligozoospermic patients was much lower than the normal group, but it was not significant.

Considering the results, the authors concluded that men, especially with severe oligozoospermia, have an elevated risk for chromosome abnormalities in their spermatozoa which might affect the fertilization and pre-embryo formation. Sperm chromatin integrity and the post-zygotic chromosomal abnormalities were considered as the probable factors involved in the pre-implantation stage embryos. Also, the authors believed that PRINS in parallel to FISH technique might allow the direct screening of a large number of spermatozoa.

In summary chromosomal abnormality studies were mainly performed with the use of the routine cytogenetic technique for patients seeking infertility treatment. The main observation in the routine cytogenetic study was involvement of inversion in chromosome 9. Few basic studies with advanced molecular cytogenetic techniques were done for aneuploidy assessment in spermatozoa. These methods have shown that the rate of aneuploidy in sex and autosome chromosomes correlate with the severity of infertility. Premature chromosome condensation of sperms was found as the main cause of failed fertilization of oocytes. 

## Yq microdeletion

The AZF region in chromosome Y, at a molecular level, and according to the certain deletion pattern of microdeletions which observed, was divided into AZFa, AZFb, and AZFc subregions (30). Further, a fourth region named AZFd, located between AZFb and AZFc was also reported. However, whether AZFd truly existed is still debatable. There are variable phenotypes regarding the deletions in different regions of AZF; as the entire deletion of AZFa region leads to SOCS (lack of germ cells in seminiferous tubules and presence of Sertoli cells only syndrome) and azoospermia (31). Complete deletions of AZFb and AZFb+c show a histological picture of SCO or spermatogenic arrest resulting in azoospermia (32).

In azoospermic men, who carry AZFc deletion, there is a chance of successful sperm retrieval, following testicular sperm extraction (TESE). In these patients, molecular genetic testing has a prognostic value for a planned TESE and genetics counselling. Since the deletion is transmissible to the male offspring, the couple should be informed about the higher risk of fertility problems in their sons. The prevalence of Y-chromosome microdeletions is approximately 1:2000 to 1:3000 males and is found to be the second most frequent genetic cause of male infertility after the KFS (33).

A wide range of variability from 1% (73) to 55% has been reported for the rate of Y microdeletion among infertile men. These microdeletions mainly occur in severely oligozoospermic men ranges from 5-10% and azoospermic men ranges from 10-15% (57-58). At present, in the case of severe male infertility, the molecular genetic diagnosis of Y-chromosomal microdeletions is done by using polymerase chain reaction (PCR); amplification of selected regions of the Y chromosome using sequence tag site (STS) primers is routinely performed as part of the infertility workup in many genetic and infertility laboratories all around the world as well as Iran, so that to give an appropriate genetic counseling and explanation for the male infertility in men with azoospermia or severe oligozoospermia (34, 35). In Iranian population, the Y microdeletion frequency, according to the databases from 2003-2012, has shown variable ranges from 5-52% which is within the range that has been reported worldwide (36-42).

Also, the range of Yq microdeletion in Iranian severe oligozoospermic and azoospermic men, regarding the results of these databases, was 5-52.6% and 5.4%-51.6%, respectively. This wide range of variability may be the consequence of different factors, such as the sample size, type of patients selection, and the variances exist among the populations; as in the paper published by Malekasgar and colleagues, the high rate of Yq microdeletion observed among the Iranian infertile men (52%) and also between azoospermic (51.6%) and severe oligospermic men (52.6%), compared to international frequency (37). 

According to the results of the investigators worldwide, deletions mainly involve AZFc, less frequently AZFb and only rarely the AZFa regions, the frequency of AZFc, AZFb, and AZFa deletions in men with Yq microdeletions is estimated to be about 60%, 16%, and 5% respectively (34, 35). It is believed that the majority of Y microdeletions arise through the distant homologous recombination between specific palindromic sequences; although, deletions generating by the non-homologous recombination were also recognized (33). Considering the results of the papers, studying the incidence of microdeletions in these regions among Iranian infertile men, there is a discrepancy among the frequency of deletions in AZF regions reported by different researchers.

**Figure 1:**
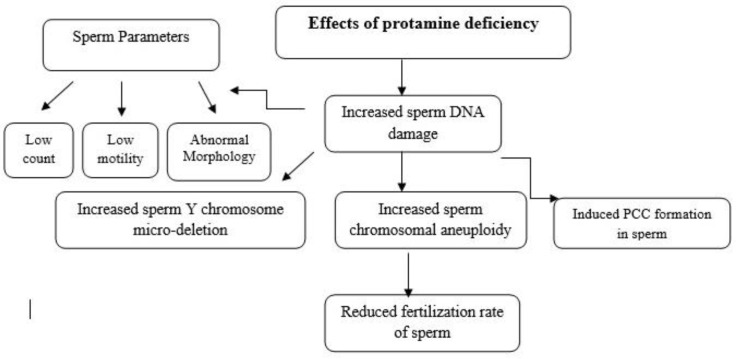
The flow diagram shows the effect of protamine deficiency and DNA damage on sperm's parameters and functions.

In a study by Omrani et al, the incidence of AZF loci microdeletion was investigated among 99 azoospermic or severe oligospermic Iranian men from West Azarbaijan. The deletions were mainly occurred in the AZFc region (87.5%) and with less or no occurrence in the AZFb (29.2%) and AZFa locus, respectively (38). Also, Keshvari and coworkers (2011) showed that microdeletions mostly involved AZFc (100%), less frequently AZFb (50%), and AZFa (25%) regions (39). The small sample size in this study might be the reason for these high frequencies since only 4 patients were found to have AZF microdeletion. In another study, Totonchi and colleagues showed among the 185 patients with AZF microdeletion 147 cases suffered from azoospermia and 38 patients from severe oligozoospermia (40). Their data indicated that the most frequent microdeletions were in the AZFc region, followed by the AZFb+c+d,AZFb+c, AZFb, AZFa, and AZF a+c regions.

Contrary to these and many other studies, in which AZFc deletion was reported to be the most frequent deletion in infertile men with Y microdeletions; in the paper published by Mirfakhraie *et al*, among 12 Iranian NOA infertile men in whom microdeletion was detected, deletion in AZFb region was the most frequent (66.67%) followed by AZFc (41.67%), AZFd (33.33%) and AZFa (8.33%), respectively (41). This was the same for the results reported by Konar and coworkers, as the frequency for AZFb microdeletion (81.81%) was much higher than AZFc region (18.18%) (42). On the other hand, although the reported frequency for AZFc microdeletion presented by Malekasgar *et al* (2008) was concordant with other studies (69.2%), for microdeletions in AZFa region the results showed higher incidences (23%) and differed significantly with many other studies (37). It seems that these discrepancies in the results might be due to geographical and ethnic variations of populations under study and differences in the patients' selection criteria and sample size as well as the number of STSs analyzed.

Different studies have shown de novo sperm chromosome instability (43-47). On the other hand, AZFc region instability following exposure to external agents, in a paper that published by Premi and coworkers showed a higher rate of microdeletion and amplification of the AZFc region and DAZ gene markers in individuals who were in exposure to the external agent as natural background radiation (47). Since many patients following exposure to diagnostic or therapeutic radiation may have different degrees of infertility. In this case, Moghbelinejad *et al* studied genomic instability of AZFc region after *in vitro* gamma-irradiated blood samples of normal, oligozoospermic, and azoospermic Iranian individuals (48). Copy number variations of studied markers in AZFc region (microdeletion and duplication) in all samples after exposure to radiation was shown; on the other hand, the frequency of instability was significantly higher in samples from infertile men compared to fertile ones. The authors concluded that this observation might be a possible explanation for induction of azoospermia and oligozoospermia after radiotherapy (48). Further studies by Mozdarani and Ghoraeian using a combined FISH and PRINS technique for detection of DAZ microdeletion in individual sperms showed considerably higher frequency of DAZ microdeletion in sperms of subfertile individuals whom their leukocytes were normal for DAZ gene (49-51).

In summary, Y chromosome microdeletion was the most studied issue of male infertility in Iran. The rate of microdeletion was found different in various geographic regions and ethnicity. However, the frequencies of microdeletions were found in a worldwide reported range with some exceptions. Few basic studied proved the influence of induced genome instability in Y-chromosome microdeletion specially AZFc region. Cytogenetic molecular techniques showed different frequency of DAZ microdeletion in sperms and leukocytes indicating that study of Y chromosome microdeletion in blood leukocytes might not represent the situation of these genes in sperms.

## Protamine deficiency

Sperm chromatin structure is highly organized and condensed, containing DNA and nucleoprotein. Protamine with positive charge constitutes a great part of nucleoproteins, which substitute histones during spermatogenesis process. There are two types of protamine in human sperm nucleus which are expressed equally in mature sperms (protamine 1 and 2) (52). There are various studies on the clinical importance of this protein. These researches showed that unequally expression of protamine 1 and 2 are obviously accompanied with male infertility or subfertility with a low number, low motility, abnormal sperm morphology, increased rate of sperm DNA damage, and influence on sperm function (Figure 1). Protamine deficiency may directly affect the fertilization process or decrease the fertilization rate, due to the co-occurrence of protamine deficiency with the late-stage spermiogenic anomalies (53-55). Also in Iran, several studies have been done in order to evaluate the impact of protamine deficiency on male infertility. In this case, Iranpour *et al* evaluated sperm protamine deficiency and its relationship with fertilization outcome post-ICSI by comparing some techniques (56). The techniques used in this study were chromomycin A3 (CMA3) staining for protamine deficiency, aniline blue staining for excessive histones, SDS for sperm chromatin stability, and SDS+EDTA for the ability of sperm to undergo decondensation. Results indicated that CMA3 was a highly sensitive and specific test for prediction of fertilization outcome post-ICSI, and CMA3 positive spermatozoa had a negative correlation with sperm fertilization rate, count, and motility; and a positive correlation with percentage of abnormal morphology. Also, Iranpour and coworkers showed protamine deficiency in sperm nucleus can cause ultra-structural anomalies in sperm chromatin such as unpacking of it (57). It is also concomitant with acrosome and sperm membrane disturbances. In another study Razavi *et al* showed that the injection of sperm into oocyte is not sufficient for sperm fertilization, and chromatin packaging can affect the fertilization rate of sperm; so, the evaluation of sperm protamine deficiency could be influential for ICSI results in procedures (58). Since CMA3 staining does not indicate the type of protamine deficiency or the P1/P2 ratio. Nasresfahani *et al* studied the expression rate of protamine 1 and 2 (or the ratio of P1/P2) and compared it with ICSI results (59). Protamine deficiency was determined with CMA3 staining, and the P1/P2 ratio was evaluated by nuclear protein extraction, acetic acid-urea polyacrylamide gel electrophoresis, and protein bands analyzed with software. Results showed a negative significant correlation of fertilization rate with protamine deficiency and P1/P2 ratio. Therefore, it was concluded that increased P1/P2 ratio affected fertilization rate and embryo quality, which subsequently might affect implantation and pregnancy outcome in Iranian population. Chromatin analysis of failed fertilized human oocytes has shown that after aneuploidy, PCC is the next prevalent cause of fertilization failure in both IVF and ICSI (59).

In other studies in Iran, the effect of sperm protamine deficiency on sperm PCC formation was evaluated post-ICSI. Results illustrated that one of the causes of the decrease in the fertilization rate of sperm, which has protamine deficiency, is sperm PCC formation in injected oocytes and there is a direct relation between protamine deficiencies of sperm with PCC formation in failed fertilized oocytes. Authors concluded that, when spermatozoa were exposed to an environment with active meiosis promoting factor (MPF), such as the oocytes in metaphase II, protamine deficient spermatozoa were more likely to undergo PCC compared with spermatozoa with a normal amount of protamine, and therefore might result in failed fertilization (60, 61). 

Studies suggest that protamine deficiency or failed oocyte activation may cause PCC formation, but it is not clear which of these two factors have more impact on fertilization failure (61). 

In order to distinguish between these two phenomena, Nasr-Esfahani *et al* ran a research, in which oocytes that failed to fertilize after ICSI were artificially activated and the association between protamine deficiency and PCC formation was evaluated in the remaining oocytes that failed to fertilize (62). The results of the study done by Nasr-Esfahani *et al* indicated that after artificial activation, post-ICSI fertilization rate increased from 59.95-87.7%, and PCC spermatozoa appeared to be present in over 50% of the remaining oocytes that failed to fertilize. The percentage of sperm PCC was significantly higher in protamine deficient samples, thus they suggested that sperm PCC which was induced by protamine deficiency after failed oocyte activation may be considered as an alternative cause of failed fertilization post-ICSI (62). 

Sperm DNA becomes susceptible to damage if chromatin packaging is not complete during spermatogenesis (63). The effect of protamine deficiency on sperm DNA damage and the relationship between these two parameters with fertilization rate and embryo development post-ICSI was evaluated by Nasresfahani *et al.* They suggested a direct relation between protamine deficiency and sperm DNA damage, and unlike protamine deficiency, sperm DNA fragmentation does not preclude fertilization. Nonetheless, embryos derived from spermatozoa with high DNA damage have a lower potential to reach blast cyst stage (64). In another study the extent of sperm DNA damage with the degree of protamine deficiency in spermatozoa of normal and subfertile individuals (oligozoospermia, asthenozoospermia, and oligoasthenozoospermia) was compared (65). Alizadeh *et al* demonstrated that there is a relationship between protamine deficiency and an increase in sperm DNA damage rate of Iranian subfertile men specially oligoasthenozoospermic patients (65). Salehi *et al* showed a negative correlation between cluster in concentration (as a seminal protein that control fertilization) with protamine deficiency and DNA damage (66).

Few investigators examined the involvement of protamine deficiency in male infertility in Iran. However, these limited number of studies clearly indicated protamine deficiency in various types of male infertility and its correlation with DNA damage.

## Sperm DNA damage

Nowadays semen analysis is regarded as a conventional test for men infertility evaluation. However, it is not possible to predict the infertility rate of sperms, because some of the factors like DNA integrity of sperm nucleus which greatly affects its fertility rate (67). Sperm DNA damages clearly lead to infertility; there are various factors resulting in sperm DNA damage, such as testicular and environmental factors. It has been shown that internal and external reactive oxygen species (ROS) are crucial factors resulting in sperm DNA damage. While a low level of ROS in semen needed for spermatogenesis, it has been shown that sperm DNA oxidation is more in infertile men than that of fertile ones (68, 69). Mozdarani and Khashai presented the first study in Iranian showing the background sperm DNA damage of 30 fertile and 90 infertile Iranian men by using Comet assay (70). The results of this study showed that background sperm DNA damage rate in sperm samples of infertile men was significantly higher than fertile ones. The highest rate of sperm DNA damages among infertile men was observed in asthenospermia sperm samples (70). Mozdarani and colleague concluded that the high rate of background DNA damage in infertile men is caused by lack of antioxidant. The results of other studies were in line with above study again emphasized sperm DNA damages in infertile and subfertile males were significantly higher than fertile ones. These studies also showed sperms with abnormal morphology and low levels of motility had more abnormal DNA damages than motile and normal sperms (70, 71). To see the relationship between the frequency of sperm DNA damage and IVF succeeding rate, Dehghani *et al* clarified sperm DNA damages by using of Acridine Orange staining method (72). Based on fertilization results, Dehghani and colleagues divided samples into three groups: less than 50% fertilization, more than 50% fertilization, and a complete breakdown of fertilization. They observed a reverse relationship between double strand DNA breakages with fertilization rate (72). They concluded that although semen analysis experiments were necessary for infertility diagnosis, it was not sufficient to predict IVF results. Based on this conclusion, it was suggested that in cases, where the rate of sperm with normal DNA was less than 47.25%, the success rate of fertilization would also be lower; so, DNA quality should be improved by proper methods before the treatment cycles (73). In this regard Fanaei *et al*, and Mardani and coworkers, showed in vitro ascorbic acid and saffaron supplementation during semen processing for ART could protect semen specimens against oxidative stress and could improve ART outcome (73, 74).

Some experiments showed that irradiation is one of the exogenous sources of ROS production and DNA damage in sperm, and causes temporary and permanent infertility. Irradiation induces sperm aneuploidy, structural chromosome aberrations, chromatin structure anomalies, DNA breaks, and higher frequency of mutations. Micronuclei (MN) are the result of chromosomal aberrations induced during the preceding mitotic division of cells. These are from acentric fragments or lagging chromosomes induced by mutagens or clastogens such as ionizing radiation, or they could be the result of non-disjunction, and so are a sign of genomic instability (75, 76). 

In this regard in a basic study by Mozdarani and Salimi and Mozdarani and Nazari indicated that when male germ cells were exposed to gamma radiation, chromosome instability expressed as chromosomal aneuploidy and micronuclei in subsequent pre-embryos. They irradiated the whole body of male NMRI mice with 4 Gy gamma-rays, and then mated them with non-irradiated superovulated female mice in 6 successive wk after irradiation, in a weekly interval. In experiments involving irradiation of both male and female mice, following irradiation, the male mice were mated with female mice irradiated after induction of superovulation. The frequency of chromosomal aberrations and MN in 4-8 cells embryos was higher in irradiated mice in comparison to control groups (76-78). Considering the fact that radiation is one of the main sources of ROS production, the effects of vitamins E and C as anti-oxidant has also been studied. Results indicated that irradiation of gonads during spermatogenesis and pre-ovulatory stage oocytes might lead to stable chromosomal abnormalities affecting pairing and disjunction of chromosomes in successive pre-implantation embryos expressed as chromosomal aberrations (76) and MN (77, 78). On the other hand, both vitamin E and C reduced clastogenic effects of radiation on germ cells leading to a reduction in the rate of chromosomal abnormalities and MN in pre-embryos; this might be due to vitamin E and C anti-oxidation and radical scavenging properties (76-78). Moghbelinejad and colleagues examined the frequency of MN in lymphocytes of infertile males after exposure to gamma irradiation. This study carried on in three groups of oligospermic, azoospermic, and fertile men. They examined micronuclei frequency in blood samples of subjects when exposed to 0, 2 and 4 Gy of radiation (79). The results illustrated a statistically significant difference between the frequencies of micronuclei in lymphocytes of infertile individuals, compared to healthy donors, before and after exposure to gamma rays (79).

Sperm DNA damage was studied both on human samples and indirectly on animals. Various degrees of sperm DNA damage were observed for subfertile males with different infertility problems. In animal studies, it was shown that induced DNA damage in sperm could pass to the next generation leading to cytogenetically abnormal pre-embryos (76-78).

**Autosomal gene mutations and polymorphisms**

Many single genes are also being investigated for possible roles in male factor infertility among the Iranian infertile men (Table I). Glutathione S-transferases related enzymes theta (GSTT1), and Mu1 (GSTM1) in addition to cytochrome P450 (CYP450) and CYP1A1 are considered to involve in the phase II biotransformation of xenobiotic drugs, poisons, and other compounds. The relationship between polymorphism of the genes which code these enzymes and male infertility was also shown (80).

Results of published meta-analysis literature reviews on the prevalence of *GSTM1* and *GSTT1* genes polymorphisms have shown that *GSTM1* and *GSTT1* null genotypes are associated with a strong and modest increase in the risk of male infertility respectively; dual null genotype of *GSTM1/GSTT1* is also significantly associated with increased risk of male idiopathic infertility (81, 82). Except for a meta-analysis paper, recently published by Song *et* *al*, which showed no association between *GSTT1* gene polymorphisms and male infertility (82).

For Iranian population, an increased risk of infertility in the patients with null genotype of *GSTM1* and *GSTT1* has been reported. Genotyping analysis of *GSTM1*, *GSTT1*, and *GSTP1* genes polymorphisms for the first time showed that combination of deletion genotypes of GST (*GSTM1* and *GSTT1*) and of *GSTM1* null, *GSTT1* null, and *GSTP1* (Ile/Ile) genotypes pose an even higher risk of infertility, but the non-deletion *GSTM1* and *GSTT1* genotypes and the variant genotypes of *GSTP1* (Ile/Val and Val/Val) have emerged as protective factors. In addition, *GSTP1* wild-type genotype in combination with *GSTM1* null or *GSTT1* null genotype increased the probability for infertility (83). The synergistic effects of the aforementioned three polymorphisms that the authors suggested were worth acknowledging. In another study by Salehi and coworker*s*, the relationship between the combination of *GSTM1* and *GSTT1* null genotypes and the increasing probability of idiopathic male infertility was emphasized again (84).

Lu and colleagues reported that *CYP1A1* polymorphisms were not involved in the etiology of male infertility (85). However, other papers showed that CYP1A1*2A CC and the combination of *GSTM1* and CYP1A1*2C genotypes were associated with increased risk of male infertility, while CYP1A1*2A TC genotype showed a non-significant increased risk of male infertility (86). In this regard results of genotyping of the CYP1A1*2A gene polymorphisms on North Iranian men with idiopathic infertility showed the frequency of TT, TC, and CC genotypes of *CYP1A1* polymorphism in the controls were the same with infertile men; authors also reported that *CYP1A1* polymorphism did not display any association with male infertility in Iranian population (87). 

Estrogen receptors (ER) are a group of proteins, found inside cells that are activated by the hormone estrogen (17β-estradiol). Two different forms of ER, usually referred to as α and β are encoded by different genes, *ESR1* and *ESR2* on 6q25.1 and 14q23.2 respectively (88, 89). Polymorphisms of the *ER* genes have been implicated in male infertility; however, there is a lack of comprehensive data probably because of interaction between genes and environment. A possible role of *ESR-α* and *ER-β* variants on male infertility in Iranian males have shown in different studies. Association study between polymorphisms of the *ESR1* (PvuII and XbaI) and *ESR2* (RsaI and Alul) genes and male infertility suggested 3 times higher frequency of the heterozygous RsaI genotype in men with low sperm concentration compared to the controls. In contrast, the proportion of homozygous AluI genotype was only 1/3 in severely oligoazoospermic men compared to control (89). Genotyping of these polymorphisms showed the presence of the *ER-α* Pvull TC, *ER-α* XbaI AG, and *ER-β* Alul GG genotypes have a protective effect on infertility, but the *ER-β* RsaI AG and *ER-β* Alul AG genotypes were associated with increased infertility risk (90). Results of a study on mutation detection in human estrogen receptor β gene among infertile male showed one heterozygous sequence variation (IVS 8-4G>A) near the 5´ splicing region of intron 8 in 5 out of 96 infertile men. No variation was identified in control population (91). The activity of androgen, an important steroid for maintaining sperm production and growth of the prostate gland, is mediated through the androgen receptor (AR), a ligand-dependent transcription factor. 

P53 (also known as protein 53 or tumor protein 53) is a tumor suppressor protein that is encoded by the *TP53* gene in humans, regulates the cell cycle, and thus, functions as a tumor suppressor. P53 has also been described as "the guardian of the genome". In humans, a common polymorphism involves the substitution of an arginine for a proline (G→C) at codon position 72 exon 4 (92). There had been no significant investigation considering the influence of this polymorphism on male infertility in other countries except only one research on China population in which no relation was observed in this regard (92). In Iran, allele frequency evaluation of this substitution in idiopathic infertile azoo/oligospermic patients and fertile healthy control men showed the presence of Arg allele among infertile men compared with controls (56% vs 44%). This research concluded that arginine allele might be at greater risk of developing idiopathic infertility in Iranian men (93).

Heme oxygenase occurs as 2 isozymes namely heme oxygenase-1 and heme oxygenase-2. The HO-1 enzyme is a stress-responsive protein which could be induced by various oxidative agents. HO-1 enzyme activity in human seminal plasma is induced by ROS which is low in azoospermia and moderate in oligospermia in comparison to normal controls (94, 95). On the other hand, several studies have demonstrated that GT-repeats in the promoter region of *HO-1* gene is highly polymorphic (96, 97). Regarding the relationship between this polymorphism and male infertility, Siasi *et al* showed that GT repeats expansion in the promoter of the *HO-1* gene was associated with oligospermia and azoospermia among Iranian infertile cases, and L allele frequency with >27 repeats was significantly higher among these group (98).

Methylenetetrahydrofolate reductase (MTHFR) is rate-limiting and plays a crucial role in folate metabolism, and it is encoded by the *MTHFR* gene (99). There are DNA sequence variants (genetic polymorphisms) associated with this gene. Two of the most investigated polymorphisms are C677T (rs1801133) and A1298C (rs1801131) (100). The results of correlation between these polymorphisms and male infertility reported in various studies are very controversial (101-103). In order to determinate the association between 3 *MTHFR* gene polymorphisms (C677T, A1298C, and G1793A) and male infertility in Iranian population, Safarinejad and colleagues showed that only C677T polymorphism was associated with an increased risk of idiopathic male infertility, and the 677T allele carriers (TC or TT) had a significantly increased risk of infertility compared with the CC homozygote’s (104). About the epigenetic study of this gene, Khazamipour and coworkers for the first time compared the methylation status of the promoter region of *MTHFR* in blood and testicular biopsies of patients with non-obstructive azoospermia and obstructive azoospermia without anomalies of spermatogenesis. In blood samples, no difference in the methylation profile of the promoter region of *MTHFR* was observed. They indicated that hyper-methylation in testis DNA from NOA patients was specific and not due to a general methylation defect, therefore, they suggested that epigenetic silencing of *MTHFR* could play a role in azoospermic infertility (105).

Glutathione peroxidase (GPx) is an enzyme family with peroxidase activity protecting the organism from oxidative damage. GPx4 has a high preference for lipid hydroperoxides and is a major seleno protein in sperm. It is also one of the enzymatic mechanisms that play multiple roles during spermatogenesis (106). According to the results of a paper which studied C-T+6, G-A +17, and G-A+1725 polymorphisms among Iranian infertile men, it was determined that the prevalence of these mutations in these infertile men was probably low, and it might have no association with the etiology of the disorder affecting sperm parameters (107). It is also worth noting that results of other studies indicated that (*GPx4*) polymorphism couldn’t generally account for the correlation of phospholipid hydroperoxide glutathione peroxidase (PHGPx) content of sperm and fertility-related parameters, but further examination of this gene as a potential cause of infertility in particular cases was warranted (108-110).

The cystic fibrosis transmembrane conductance regulator (*CFTR*) gene is located in region q31.2 of human chromosome 7. The normal CFTR protein product is a chloride channel protein found in cell membranes of lung, liver, pancreas, intestine, reproductive tract, and skin. Defective versions of this protein, caused by *CFTR* gene mutations, can lead to the development of cystic fibrosis (CF) and congenital bilateral aplasia of the vas deferens (CBAVD) (111). CBAVD is a condition present since birth in which the tubes that carry the sperm out of a man's testes (the vas deferens), fail to develop properly which can cause male infertility. To investigate CBAVD at the molecular level in Iran, Radpour and colleagues have characterized the mutations in the *CFTR* gene in patients with this condition; none had clinical manifestations of cystic fibrosis (CF). They analyzed a DNA variant (the 5T allele) in a non-coding region of *CFTR*, which causes reduced levels of the normal CFTR protein and M470V exon 10 missense polymorphism. Their results showed that the combination of the 5T allele in one copy of the *CFTR* gene with an F508del mutation in the other copy was the most common cause of CBAVD in Iranian patients (111).

Different alleles at the (TG) m (T) n polymorphic locus at the 3' end of human *CFTR* intron 8 determine the efficiency of exon 9 splicing. It was shown that among Iranian CBAVD men, longer TG polymorphic tracts increase the proportion of exon 9 transcripts deletion, but only when it was activated by the 5T allele; highest level of exon 9+ splicing efficiency was among the tested samples with the (TG) 12 (T) 7 allele (111). The first NBD (NBF1) plays an important regulatory role in CFTR function (111). In another study, one novel nonsense mutation (K536X) was detected in the NBD1 region, and considered as a severe allele responsible for elevated sweat chloride levels and obstructive azoospermia. Two other novel missense mutations, not reported previously, were (Y122H and T338A) in the M2 and M6 regions of *CFTR* gene (112).

There have been numerous investigations that presented a significant relationship between reduced protamine genes expression and male infertility. On the other hand, studies on the variations in human protamine genes in different populations have indicated that these variants in protamine genes are population-specific, and except a few studies, most papers have reported that there is no specific relation between protamine gene variants and male infertility (113). Esfahani *et al* performed the first study in this regard, where two single nucleotide polymorphisms (SNP) in protamine 1, 2 genes were investigated. G197T in protamin1 gene converts argenine to serin; it also causes phosphorilation and incorrect protamine-protamine interaction. Second SNP was C248T in protamine 2 gene which causes immature stop codon and truncated protein. They studied SNPs on blood samples of 273 oligosperm and 35 fertile men by using RFLP technique. None of the reported SNPs were observed in all 308 samples (fertile and infertile). They concluded that in Iranian population it was not possible to use these two SNPs as detecting markers (114). Siasi and coworkers, studied the relationship among some protamine genes family SNPs including *PRM1* (C321A), *PRM2* (C248T), *TNP2* (T1019C), G1272C, and G del in 1036 and 1046 bp. No polymorphisms were found for tested SNPs except for *PRM1* (C321A) and *TNP2* (G1272C), in which frequency of altered AA and GG genotypes were slightly higher in the infertile case group (115). These results were consistent with previous studies and indicated that all tested SNPs were not associated with oligospermia, azospermia, and idiopatic male infertility in Iranian population. Some other polymorphisms are summarized in table II.

Follicle stimulating hormone (FSH) is essential for normal reproductive function in males and females. FSH acts through its specific receptor named *FSHR* which is expressed only in Sertoli cells in humans. Among several SNPs within the *FSHR* gene, A919G and A2039G affect the receptor function (116). Up to now, controversial findings have been obtained concerning the effect of SNPs within *FSHR* gene on male infertility. In this regard, Gharesi-Fard and colleagues showed, among the Fars population, two polymorphisms of this gene (A919G and A2039G) might increase the susceptibility to obstructive azoospermia. They recommended further investigations among the other ethnic populations of Iran (117).

YBX2 is the human homologue of Xenopus DNA/RNA-binding and mouse MSY2 proteins located on chromosome 17p13.1. (118, 119). Studies with animal models showed expression of this protein in meiotic and post-meiotic germ cells. Loss of its expression leads to the nuclear condensation defects that occur in Msy2-null late-stage spermatids (120). Some polymorphisms of this gene associated with male infertility in diverse ethnic populations were shown (121). About the Iranian population, in one study, Najafipour and coworkers observed down-regulation of this gene in testis tissue of non-obstructive azoospermia men. In terms of pathological evaluation, these patients had spermatid maturation arrest (122).

YBX2 acts as a mRNA stabilizer and a transcription factor of *PRM* genes; and, its loss of expression is likely to contribute to the nuclear condensation defects in Msy2-null late-stage spermatids (123). Moghbelinejad *et al *evaluated the relationship between PRM deficiency and YBX2 expression level in testis tissue of azoospermic men. They showed a significant correlation between PRM2 mRNA deficiency and a lower YBX2 mRNA content in testicular spermatids of infertile men. They concluded that these molecules may be regarded as suitable predictive biomarkers to discriminate between fertile and infertile men (124). In another study, Najafipour *et al* evaluated exon 1 of *YBX2* gene polymorphisms frequency, in Iranian infertile men. Their results showed, among the different polymorphisms of this gene, the frequency of TT genotype in rs222859 G>T polymorphism, was significantly higher in azoospermic samples in comparison to normal ones. Gene expression study of this gene showed, downregulation of *YBX2* gene in blood and testis samples, but there was not a significant difference in gene expression level between patients with mentioned mutation and without the mutation. They concluded that in future studies, it is better to investigate the effect of this mutation on the 3D structure of the protein (125).

In essence, various autosomal gene mutations and polymorphisms were investigated for their possible role in male infertility. All of the genes studied were mainly reported in the literature but these studies verified their impact on Iranian individuals. 

**Table I:** Known and reported autosomal genes mutations and polymorphisms in Iranian infertile men

**Study**	**Gene name**	**Types of mutation and polymorphism in Iranian infertile men**
Plaseska-Karanfilska D, *et al* and Shefi S, *et al* (43, 44)	Glutathione S-transferase theta & mu (*GSTT1*, *GSTM1*)	Null genotype
Omrani MD and Nordenskhold A (91)	Androgene receptor (*AR*)	CAG repeat in codon region (26 length), 1510CRA transversion in exon 1
Omrani MD, *et al* (89)	Estrogen receptor ß (*ER2*)	RsaI AG Alul AG (IVS 8–4G>A): near the 5’ splicing region of intron 8
Mashayekhi F and Hadiyan SP (93)	*P53*	(G→C) at codon position 72 exon 4
Siasi E, *et al* (98)	Heme oxygenase 1 (*HO1*)	GT repeats expansion in the promoter (27 length)
Khazamipour N, *et al* (105)	Methylenetetrahydrofolate reductase (*MTHFR*)	C677T (rs1801133)
Radpour R, *et al* and Hojat Z, *et al* (111, 112)	Cystic fibrosis transmembrane conductance regulator (*CFTR*)	5T allele in a noncoding region M470V and M469I in exon 10 (TG) 12 (T) 7 repeat in splicing site of intron 8 (K536X) in the nucleotide-binding domain 1 (NBD1) (Y122H and T338A) in the M2 and M6 regions
Asadpor U, *et al* (130)	Ubiquitin-specific protease (*USP26*)	370-371insACA, 1423C > T and 494 T > C.
Sarkardeh H, *et al* (131)	Mov10 RISC complex RNA helicase-like 1(*MOV10L1*)	Missense mutation G→A (rs2272837) Nonsense polymorphisms C→A (rs2272836), A→G (rs11704548), C→T (rs138271) in the exonic sequences. C→A (rs12170772), G→A (rs2272840), A→G (rs17248147) in the intronic sequences.

**Table II:** The frequency of AZFa, b, c microdeletions of Y chromosome reported for the Iranian infertile and subfertile men

**Study**	**Studied markers**	**Y microdeletion frequency** *
Asadi F, *et al* (36)	AZFa: sY81, sY83, AZFb: sY127, sY130, sY131, sY147, sY149, sY157, sY158, AZFc: sY254, sY276	Two of the patients (5%), in AZFc region (DAZ locus)
Malekasgar AM, *et al *(37)	AZFa: sY81, sY83, sY121, AZFb: sY128, sY130, sY133, sY143, AZFc: sY147, sY149, sY242, sY231, sY254, sY255, sY182, sY238 sY202, sY158, sY157	AZF: 24.2%, AZFc: (87.5%), AZFb: (29.2%), AZFa: (0%)
Omrani MD, *et al *(38)	AZFa: sY121, sY182, sY90, sY87, sY86, sY84, sY81 AZFb: sY134, sY133, sY130, sY128, sY127, sY124, sY117, sY109, sY11 AZFc: sY272, sY255, sY254, sY151, sY158, sY157, sY155, sY146, sY283, sY277, sY238, sY276 AZFd: sY152, sY153	AZF: (52%), AZFa: (23%), AZFb: (23%) AZFc: (69%)
Mirfakhraie R, *et al *(39)	AZFa: sY81, sY84, sY86, AZFb: sY121, sY124, sY127, sY134, AZFc; sY242, sY239, sY254, sY255, AZFd: sY145, sY153	AZF: 12%, AZFa (8.33%), AZFb (66.67%) AZFc (41.67%), AZFd (33.33%)
Keshvari SM, *et al *(40)	AZFa: SY121, SY83, AZFb: SY121, SY134 SY 143, AZFc: SY254, SY255, SY149, SY202, SY231.	AZFa: (25%), AZFb: (75%), AZFc: (100%)
Totonchi M, *et al *(41)	AZFa: SY84, SY83, AZFb: SY142, SY134 AZFC: SY157, SY154, SY158, SY254	AZFa (2.16%), AZFb (4.32%), AZFc (51.35%), AZFa+c (0.54%), AZFb+c (15.67%), AZFa+b+c (15.67%)
Konar E, *et al *(42)	AZFa: SY182, AZFb: SY133AZFC: SY255, SY254, SY146, SY158, SY238, SY155, SY277, SY272, SY283, SY157, AZFd: SY153	AZFa (-), AZFb (20%), AZFc (80%), AZFd (-)

* Percentages given in the table are derived from the stated reference.

**X-linked genes polymorphisms and mutation**

It is estimated that 10%-20% of patients with male infertility could have reduced AR function as a result of CAG repeats. The expansion of CAG repeats in infertile men was shown in some studies, while other studies have not (126). In one study, 13 different alleles in the infertile group ranging from 18 to 32 CAG repeats and 11 different alleles in control groups ranging from 16 to 28 CAG repeats have been reported. The mean length of CAG repeats was significantly different between infertile and fertile groups. Long androgen receptor CAG allele, which was found in up to 38% of infertile males, was associated with defective spermatogenesis (26). The average CAG repeat length in exon 1 of the *AR* gene found in other white populations was similar to the studied Iranian population (127). The results of the point mutation analysis in this gene showed 8 mutations in patients with azoospermia, 4 of which were located in exon 1. In a case report study by Mirfakhraei and colleagues, results of sequencing analyses detected a 1510CRA transversion in exon 1 of the *AR* gene, which resulted in a p.Pro504Thr substitution in the transactivation domain of the protein. This substitution has not been previously reported. Authors also suggested molecular analysis of this gene in Iranian infertile men (128). 

Ubiquitin-specific protease 26 (*USP26*), is an X-linked gene involved in spermatogenesis (129). USP26 express abundantly in mice testis. USP26 belongs to a family of de ubiquitinating enzymes (DUB), which play an important role in various cellular processes including growth control, differentiation, oncogenesis and genome integrity (129).

Sequence alterations in the *USP26* gene were shown in populations of men with severe male factor infertility; including men with Sertoli cell-only syndrome (SCO) and maturation arrest. Three mutations usually found to be clustered in the same allele, 370–371insACA, 494 T>C and 1423C>T (130). Another study in Iran, indicated that there was a haplotype between three observed mutations in Iranian population. Surprisingly, the total frequency of mutations in men with a history of idiopathic recurrent pregnancy loss and azoospermic cases was significantly higher than that of in control groups (131-132).

In summary, X-linked genes polymorphisms and mutations have been studied in a limited number of investigations. Results of these studies were nearly similar to reported findings in the literature.

## Conclusion

During the last 17 yr, great efforts have been made to investigate the molecular genetics and the cytogenetic basis of male infertility in Iran. The studies being done involved various aspects of genetic studies from molecular to cytogenetic of male infertility. Most of the investigations were novel in their nature worldwide and improved our understanding of male infertility. Although compared to extensive research has been done worldwide on the issues discussed, the contribution of these publications adds little to the literature. However, all the studies being done on Iranian patients shed some lights on the genetics of male infertility and paved the way for further investigations. 
